# Suitable Recreations for Dentists

**Published:** 1899-11

**Authors:** M. Cavanagh

**Affiliations:** Owen Sound, Ont.


ARTICLE VI.

### SUITABLE RECREATIONS FOR DENTISTS.

M. CAVANAGH, D. D. S., OWEN SOUND, ONT.

It will not be the object of this paper to go very deeply into the scientific aspect of this subject, but to bring before the convention and the members of the profession at large a few plain facts with regard to the subject of recreation, and the vital importance of properly disposing of our spare time or "off hours" from the toils of the office, that we may obtain rest from our daily occupation and at the same time develop our faculties along other lines than those of our profession, so that in the end we may bring greater strength, clearer judgment, and better skill to the service of those who repose confidence in us, and that we may be more worthy of that trust.

It is a subject that in my mind has not had the prominence in our dental literature and dental education that its importance deserves. We find a superabundance of theories on pyorrhea, on treating and filling nerve canals, on crowning and bridging; but on the subject of *recreations* (which I feel safe in asserting is just as important to the dentist as any of the above, unless he has great faith in the reward that awaits the martyr), we find very little information. The reason for this lack of attention to this particular subject no doubt originates in the old idea that education consists in book-learning, and that time spent on other than our life vocation is time wasted, and a great many members of our profession are living up to this old and exploded theory to the letter.

In dealing with the subject of recreations in this paper we will make use of the term in its broadest sense. The word is derived from the Latin "creo"—I make, and the prefix "re," meaning again, the literal meaning of the word being to make again, to build up or renew; therefore any agent, any exercise or pastime that aids in invigorating or developing us physically or mentally may properly be called a recreation. One writer on this line says that our first aim and object should be to be men, and, second, to be dentists. Therefore every agent that assists in building up a strong, energetic, well-rounded manhood is of necessity a recreation, and all recreation should be indulged in with this worthy object in view of improving ourselves. It is therefore an educational process, and may overlap or be overlapped by some other papers on the programme.

The dental profession is known by all present to be exacting both mentally and physically—exacting on the mind owing to the fineness of the work and the close application and attention to the most minute details necessary to insure even moderate success; wearing, physically, owing to its confinement, to the long hours spent in one position —and very often an unnatural position at that—and to the undue length of the day often necessary to keep even with our work; trying both mentally and physically, owing to the frequent and harrowing ordeal of spending hours on nervous, irritable patients, who exact their "pound of flesh," and a greater proportion of patience and nerve force every time they call on their dentist. Therefore, just in proportion to the amout of physical, mental, and nervous strain we undergo, just in that proportion does our system demand counteracting and recreative influences, and just so sure as these influences are neglected, just so sure are we on the gradual decline that leads to ill-health and all that it entails, viz., loss or professional skill, loss of practice, loss of income, loss of happiness, in fact loss of the ideal we sacrificed health in trying to maintain; simply killed "the goose that laid the golden egg," and yet how many are committing this gradual suicide. Comparatively few professional men, and especially dentists, utilize their hours of recreation intelligently with reference to the nature of their daily and the compensating character of the exercise necessary in order to maintain mind and body in normal and healthy condition.

It is an axiom in the active life of the word, "That to the young all things are possible," meaning simply that with youth and its adjuncts, health and strength, we should all obtain an enviable position in whatever line our occupation may be. In order to accomplish this pre-eminence, health is the first essential. We find practical illustrations of this fact by looking back over the intellectual lights of the world, and find that the most brilliant intellects that maintained their power for any considerable length of time were supported by strong and vigorous bodies, so we conclude that the maintenance of health is of utmost importance if we are to excel in our chosen profession.

Health is the greatest of temporal gifts. It is an essential to the faithful and competent discharge of duty in every walk of life, and in this age of keen competition it is almost a neccessity to success, and each of us has the obligation devolving upon us of maintaining in as great a degree of perfection as possible this priceless boon. It is immeasureably of more importance than any gain that may be obtained from its sacrifice.

It is also one of our best advertisements; the dentist with healthy, robust frame has a great advantage over his equally skilful brother practitioner in a languid or emaciated body from the standpoint of personal attraction alone, and this certainly counts for something in our profession; besides no matter how strong the intellect or skilful the hand may be, it can never be at its best or render the same service when laboring under the disadvantage of ill-health that it could if supported by physical strength. The happy possessor of health and strength will accomplish more work, do it better, and do it with more pleasure to himself and satisfaction to his patients then he otherwise could do.

So viewing this subject from any and every standpoint we can but come to the conclusion that the obtaining and maintenance of health and strength is our first duty, and should be an important portion of our education.

Physical exercise or recreation carefully and judiciously indulged in is the recognized fount from which springs health and strength. "Shun drugs as you would a plague" are the wise words of one authority on this subject.

As health and strength are the first and most important factors in perfectly developed manhood, we shall first dwell upon the recreations best adapted for physical development and culture and their action on the human frame and organs.

Admitting the value of health and strength, then we may ask, what is health and strength, and how are they to be obtained and maintained by any system of exercise?

Health consists in such a condition of growth and development of the organs of the body as enables them to fulfil the functions easily and completely, and to resist effectively attacks of disease; therefore, it includes in its meaning a certain degree of strength, and strength properly obtained is in the highest degree conducive to health.

Before discussing fully the best methods of recreation for physical development, it will be necessary to take into consideration certain elementary physiological facts that we may more readily see the beneficial effects of exercise.

The life of the body as a whole depends upon the life of innumerable atoms which constitute it and which are continually dying, being cast off and replaced by others, and the general health depends directly upon the activity of this recreative process and the perfection with which it is carried on. The blood carries to every tissue and organ of the body the food necessary for its repair, growth or development. If we move a hand or take a step, certain cells or atoms die and are disintegrated as a result of that movement, new cells must be supplied to take their place and the old ones must be removed and carried to organs whose functions it is to eliminate them from the system. All this is done by the blood which, however, becomes loaded with effete material, much of which is thrown off by the lungs in the form of carbonic acid gas.

The health and strength of any individual are in direct proportion to the thoroughness and celerity with which these broken down cells are removed from the system and replaced by others, and consequently anything which promotes the activity of this process is a beneficial and healthy recreation. The only means for safely and continuously stimulating this process into increased activity is physical exercise.

Exercise may be defined as *muscular contraction*, and acts in a manner readily understood. All movements are made by such contraction. If we raise an arm, take a step or bend a finger, we accomplish it, because in response to our will, certain muscles or sets of muscles contract. This is true of all voluntary movements. Other sets of muscles, of which the heart is an example, are not controlled by the will, but act in response to other stimuli. The stimulation of the muscles of the heart is produced by the pressure of blood itself, particularly the venous blood which is brought back from the tissues laden with carbonic acid.

As soon as any act of exercise is begun, a number of the voluntary muscles are put into action. Their contraction compresses the blood vessels and impels the venous blood actively towards the heart, which thus stimulated, vigorously sends the blood in large quantities to the lungs. Then the inspiratory muscles contract and lift the frame of the chest, enlarging it both laterally and antero-posteriorly; the diaphragm pushes down the contents of the abdomen and air rushes into the chest to fill the space thus produced and supplies the oxygen necessary for the purification of the blood; this is then returned to the heart to be distributed anew throughout the system, carrying with it the materials needed to supply the waste caused by the muscular movement originally made. These materials, with regular and systematic exercise, are deposited in larger quantities than are required to counterbalance the destruction which has taken place, then we have the muscles growing or increasing in dentistry or both.

The involuntary muscles also, including the heart and diaphragm, grow stronger in the same manner, the pulsations of the heart becoming more forcible with exercise, but at the same time slower and less obtrusive, showing that it accomplishes its work more easily.

The increased activity of the circulation carries the blood in larger volume, not only to the muscles, but also to all the organs of the body and thus stimulates them to greater activity, strengthening the appetite, digestion and nutrition, thus causing a gain in weight.

The lungs expand more fully and completely and take in an increased quantity of air, thus improving the respiration. The larger amount of blood sent to the skin increases perspiration which carries with it much of the worn-out and useless material of the system through the pores of the skin adds to the resistive power against evil influences from without, such as bad air, etc. The bony framework of the chest, though elastic, does not go quite back to its original dimensions, but gradually increases in size, giving additional room for the important organs which it contains and protects. Thus we find that physical exercise is a recreation indeed, and is essential to the body if we are to keep it in a vigorous, healthy state, for it is by *use* that we develop.

It is true that the voluntary muscles that are the first cause of the action are benefitted to the greatest extent, but at the same time we add to the functional ability of the involuntary muscles, while through the process of respiration and circulation we influence not only the health and strength, but also the growth and development of the whole body, and thus we find in various physical recreations, the secret by which any part of the physique may be strengthened and developed.

The rule of health which prescribes exercise is most easily transgressed, to violate it is only to disregard it, and a sin of omission is always easier than a sin of commission. All glaring sins of commission bring a direct and traceable result. Intemperance, gluttony, and dissipation of all kinds speedily bring a penalty with it, but the evils due to the want of recreation, though no less serious, are insiduous and elusive, and, when we feel the result, in languidness, want of energy, etc., tonics, stimulants and drugs are generally called upon to produce results which would be far more radical and permanent, were we to follow out nature's method, and systematically and moderately indulge in suitable physical culture. We have in this the remedy for most of our ills.

While physical recreation is invaluable in regulating the system and preserving its tone and vigor, care is necessary in prescribing it, and indulgence must be tempered with wisdom and judgment or it is sure to defeat its own end. Our national sports are beneficial, and can only be recommended when participated in with moderation. The desire should be not to excel along any practicular line of athletics, as excellence means over devolment in this age of professionalism and should be discouraged. The professional athlete, while appearing to be a model in physique and the picture of health, is, as a rule, short-lived, owing to the vitality being consumed or the vital organs strained in the over-production of some particular set of muscles, rather than the moderate development of all the organs and muscles of the body.

There can be no fixed rule laid down for the taking of exercise, as each constitution differs in its demands, and it should be the aim of each individual to discover their physical weakness and to patiently and persistently endeavor to bring their debilitated organs or faculties up to the standard. Wonders can be accomplished by patience and perseverance, Sandow, Sampson and Cyr, men who have astonished the world with their feats of prodigious strength, are just as much marvels of patient, untiring effort in their training as they are marvels of strength. We do not make use of this illustration with any intention of its being an incentive to imitate these men, but mere examples of what can be accomplished in physical development, and that even the weakly and delicate may be sure of the result if they persevere in moderate exercise.

While there can be no hard and fast rule for our guidance in this matter a few general principles may be of service in bringing us to a decision as to what kind of exercise is beneficial in our case.

1. Individuals with weak heart, lungs, or in fact, any of the vital organs, should not participate in any very violent or exciting recreation, or the result will be fatigue and weakness; much the same effect as is produced by overwork; mild exercise, gradually increased, must be the rule for such, if the effects are to be beneficial.

2. Those of strong physique demand more vigorous exercise; but their natural tendency is to develop along the lines they need it least; having strong arms and back they adopt rowing, weight athletics, etc., which further develop these already powerful sections of the frame instead of bringing the weaker portions up to this standard of excellence. Those with powerful lower limbs naturally take to walking, running, jumping, bicycling, whereas the muscles of the arms and chest should receive special attention.

3. Those of highly nervous temperament require plenty of sleep and abundance of fresh air. Sleep alone is said to be the secret of Gladstone's wonderful power of endurance; other instances are on record where people of great business capacity found one day each week spent in sleep necessary to keep the mind fresh and vigorous and the nervous system equal to the strain, and yet, if any part of the day has to be shortened to make more room for work it is generally the hour of sleep, but always to our injury.

4. Those having a natural fondness for athletics should be guided and even restrained, while those of more sluggish temperament and studious habits require to exercise will power and force themselves into a course of physical culture as the only remedy for a languid, listless frame; a renewal of our energies is not brought about by idleness, laziness or dissipation; it is use that hardens muscle, develops intellect and gives freedom from that sense of fatigue, that is the portion of those who lapse into indifference mentally and physically, or degenerate into mere "money makers."

Second only in importance to physical recreation is the culture and development of the intellect, and as physical activity is necessary to physical strength and endurance, so is mental activity essential to advancement in the realm of knowledge.

It is not enough that we should know a great deal about our profession; no matter how well posted or how perfectly developed we may be along one line or channel, we are of necessity narrow minded and somewhat of a bore to the society in which we are placed, unless subjected to the broadening influence of study and culture along other lines.

This can only be accomplished by those engaged in professional duties throughout the day by judiciously occupying their hours of recreation. It is this intelligent use of spare time that constitutes the difference between the recreation of a man and the rest of an animal. These hours, though somewhat limited, are not to be despised. Many a man has become famous throug carefully employing them with a definite object in view. It is the intelligent use of these hours that prevents us degenerating into cranks and old fogies, and falling into grooves and ruts in our professional life.

As an illustration of what the want of educational influence and the necessity of progression will accomplish, I wish to relate a personal experience with a member of our profession who lives less than 100 miles from the City of

Toronto. He was a stranger to me, but I called on him when passing through his town, and had not been in his presence a quarter of an hour before I found out I had struck the fountain-head of dental knowledge. Crown and bridge work were accomplishments of his when the Dental College was struggling with the alphabet of dentistry; in fact, this worthy institution, from the dean down, was a huge swindle, and all that was necessary to make a brilliant success as a dentist, was to possess the same amount of brains that he possessed, and use them as he did, and then dental colleges, conventions and literature were a needless expenses. It is needless to state that this dental headlight is only such in his own estimation.

This is perhaps an exaggerated instance of bigotry and narrowmindedness, but we certainly, one and all, descend the plane to a certain extent that leads to this goal, unless we take advantage of the opportunities we have to broaden our sphere of comprehension, and brighten our ideas by learning from those who have had more experience than we.

While recreation along other lines is most essential, we should not exclude entirely the subjects that relate to our profession.

One of the very best and most profitable means of recreation is the annual and local conventions. In addition to the papers read, discussions engaged in, and information received, there is the indefinable satisfaction of contact with numbers of men who are engaged in the same occupation; the renewal of old acquaintances and friendships; difficulties explained away, and many other benefits derived from thus meeting and indulging in professional intercourse, and while it is somewhat along the line of our office work, it is so entirely different in its relations and surroundings, that it affords a complete rest, so that at the end of the vacation, we return with renewed health, brain rendered more active, and a sense of weight removed which continues as an incentive and inspiration through the balance of the year.

One essential to an ideal mental recreation is that it must be of absorbing interest, such that it will take our minds and attentions entirely from the worries and responsibilities of our office-work, giving the faculties thus employed a complete rest, and at the same time, developing others not thus employed.

It should be entertaining and instructive, something for which we have a natural inclination, and which attracts us toward it rather than requires compulsion on our part, for anything that becomes a labor adds to the fatigue of the day rather than an aid in recuperation.

Another essential in our choice should be the value of the subject as an education. It is a very easy matter to fritter away our spare moments in a light, frivolous kind of reading or other pursuit, perhaps pleasurable in its character, and to a certain extent, restful from our daily toil, but from which we obtain no lasting benefit, and which is in reality injurious in that it dissipates the mind, makes it less retentive, and destroys a taste for that which is weightier and worthy of our consideration.

Still another object should be to pursue each subject chosen (and they should be limited to correspond with the time at our disposal) until we acquire more than a passing knowledge of those undertaken that we may fit ourselves for usefulness outside our profession as well as in it. Great good has been accomplished, and many men made famous by properly used hours of recreations.

All of our possibilities do not lie in the one sphere that we have chosen for our vocation. We should not narrow ourselves down to the one idea of life; we are gifted with many capabilities, and we are not filling the place in the world that we were designed to occupy unless we develop and then use, as far as possible, our various talents with the ultimate end in view of doing good.

We can only make mention of very few of the many subjects that, in our mind, might be pursued with profit and pleasure during our hours of recreation. These, of necessity, must vary with the tastes and capabilities of the individual, and as no definite rule could be laid down in the realm of physical culture, neither can we in the mental; any at all, when properly used, may be, and, no doubt are, beneficial to a certain extent, but our aim should be to pursue those which yield the greatest amount of good.

Music, I consider an ideal recreation; it is always pleasureable and restful, refining in its influence, elevating in its character, and boundless in its possibilites, a knowledge of which might well be coveted by all.

Botany, with its health-producing rambles through wood and meadow, in search of rare plants, is a pastime worthy of consideration, instructive and interesting, with the double advantage of being both a physical and mental recreation.

Amateur photography is still another recreation that, at the present time, is already a great favorite—and justly so in cultivating a desire for art, in enabling us to see beauties in both art and nature, to which we were formerly blind, in adding still another ray of pleasure to our lives.

Electricity, with its ever-widening influence is a fruitful theme of thought, and especially interesting, in that it is being so extensively used in our profession.

Thus we might proceed, *ad infinitum*, but sufficient has already been said, and with again trying to impress upon the members of the profession the absolute necessity of making spare time, taking it from hours that may seem to be lost, and then using them in a manner that will recuperate mind and body, we gain immeasurably in health, happiness, and in the end, financially as well.—*Dominion Dental Journal.*

